# Supplementary material for: Novel Tetrahydroisoquinoline-Based Heterocyclic Compounds Efficiently Inhibit SARS-CoV-2 Infection In Vitro
Source: Viruses. 2023 Feb 11;15(2):502. doi: 10.3390/v15020502 (PMC9965753; doi:10.3390/v15020502)
Supplement: Supplementary file 1 [file viruses-15-00502-s001.zip › viruses-2198848-supplementary.pdf]

Supplementary materials for

Novel tetrahydroisoquinoline based heterocyclic compounds efficiently inhibit SARS-CoV-2 infection in vitro

Xi Wang [1,†], Nikola T. Burdzhiev [2,†], Hengrui Hu [1], Yufeng Li [1], Jiang Li [1], Vesela V. Lozanova [3], Meglena I. Kandinska [2,*] and Manli Wang [1,4,*]

1 Center for Biosafety Mega-Science, Wuhan Institute of Virology, Chinese Academy of Sciences, 430071 Wuhan, China
2 Sofia University "St. Kliment Ohridski", Faculty of Chemistry and Pharmacy, 1, James Bourchier avenue; 1164 Sofia, Bulgaria
3 Department of Medical Chemistry and Biochemistry, Medical Faculty, Medical University-Sofia, 2 Zdrave Str., 1431 Sofia, Bulgaria
4 Hubei Jiangxia Laboratory, 430071 Wuhan, China
* Correspondence: ohmk@chem.uni-sofia.bg (M.I.K); wangml@wh.iov.cn (M.W)
† These authors contributed equally to this work.

Table of Contents

Spectra of *tert-butyl rel-4-(((3R,4S)-3-(1H-indol-3-yl)-1-oxo-2-propyl-1,2,3,4-tetrahydroisoquinolin-4-yl)methyl)piperazine-1-carboxylate (**1**)* 2

Spectra of *rel-(3R,4S)-3-(1H-indol-3-yl)-4-(piperazin-1-ylmethyl)-2-propyl-3,4-dihydroisoquinolin-1(2H)-one (**2**)* 4

General structure of compounds *trans-3 - trans-9* 6

Spectra of *tert-butyl rel-4-(((3R,4S)-3-(1H-indol-3-yl)-1-oxo-2-propyl-1,2,3,4-tetrahydroisoquinolin-4-yl)methyl)piperazine-1-carboxylate* (**1**)

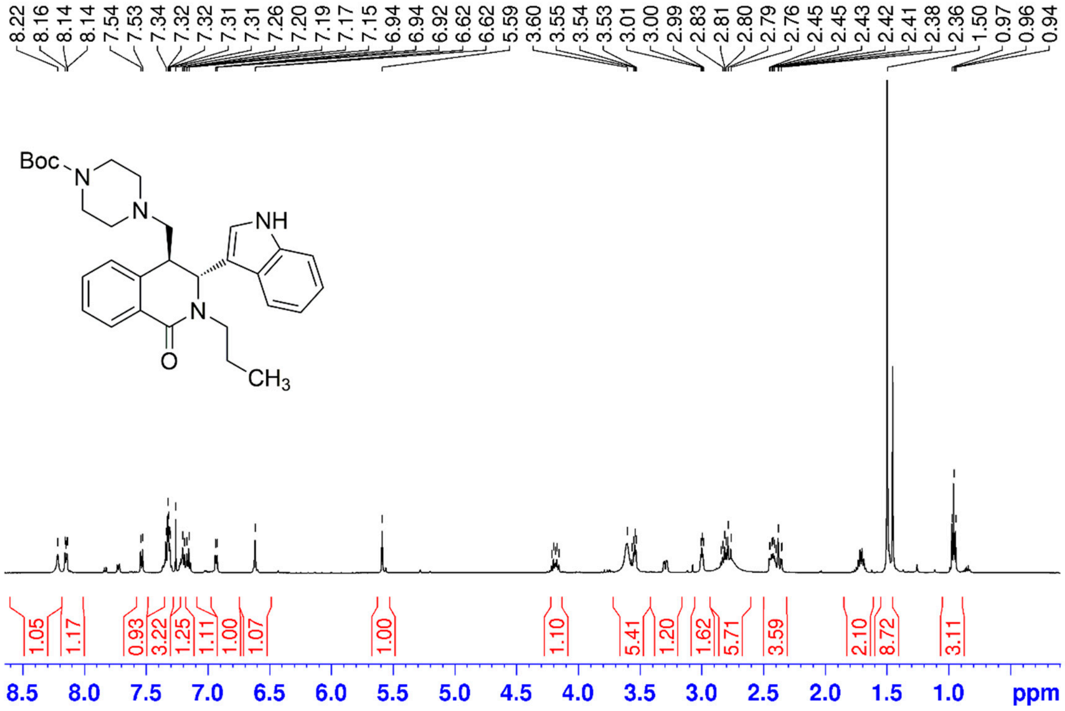

Figure S1. ^{1}H NMR (DMSO-d_6) of compound *trans*-**1** at 298 K.

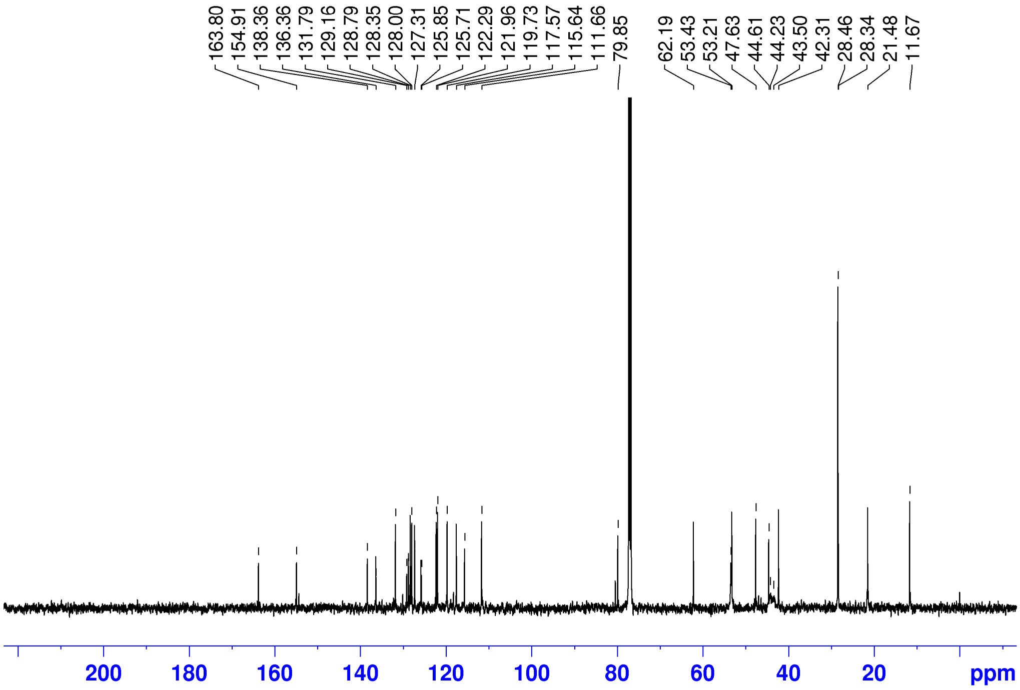

Figure S2. ^{13}C NMR (DMSO-d_6) of compound *trans*-**1** at 298 K.

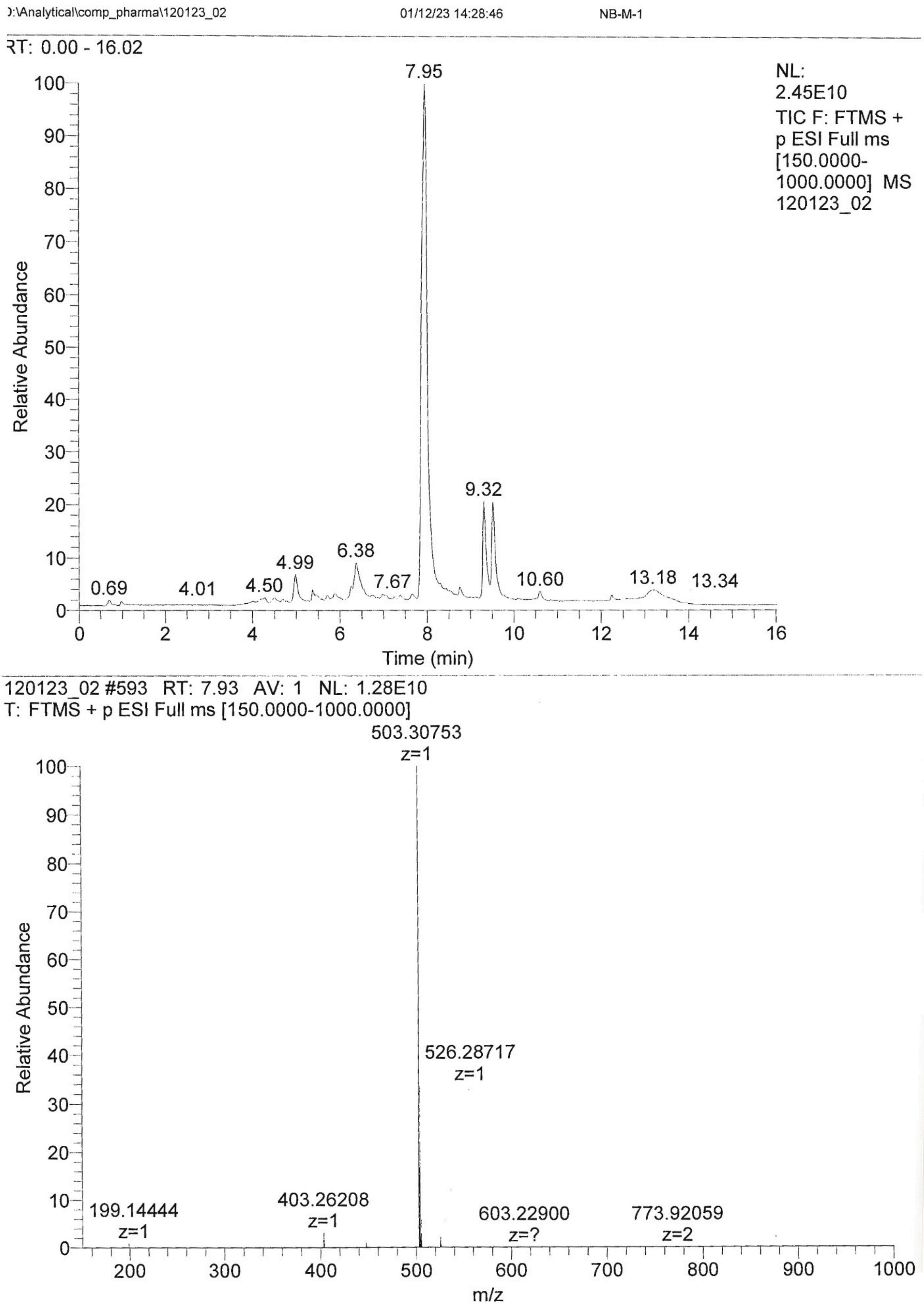

Figure S3. HRMS spectrum of compound *trans*-**1**.

Spectra of *rel-(3R,4S)-3-(1H-indol-3-yl)-4-(piperazin-1-ylmethyl)-2-propyl-3,4-dihydroisoquinolin-1(2H)-one* (**2**)

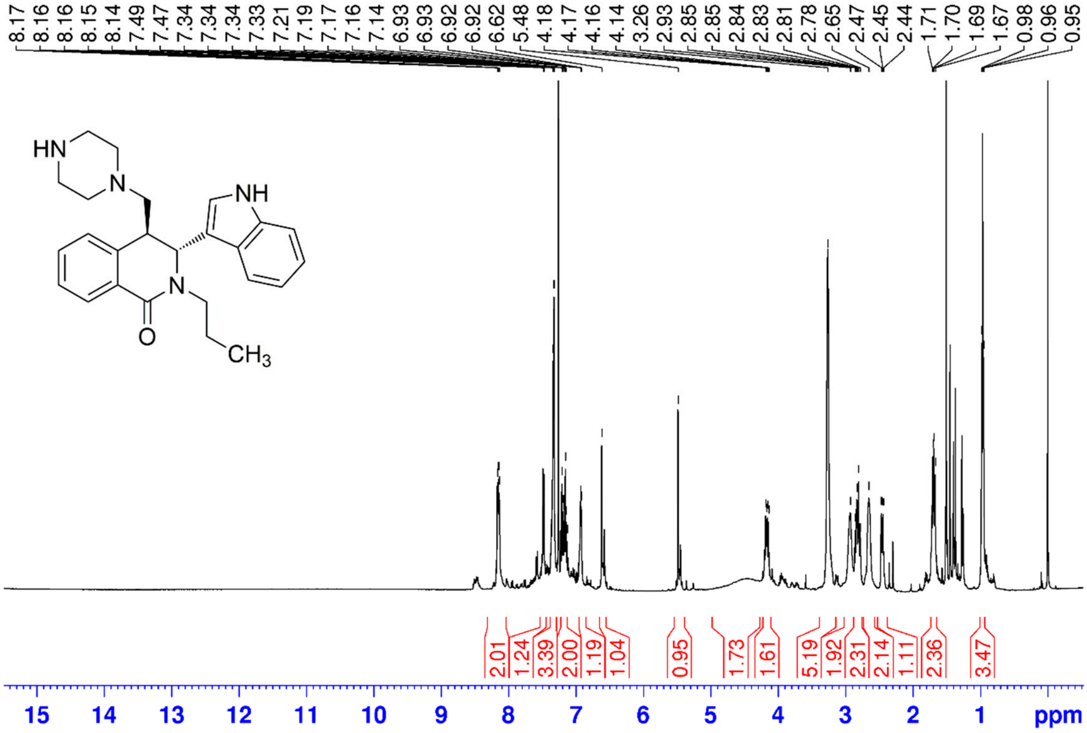

Figure S4. ^{1}H NMR ($CDCl_3$) of compound *trans*-**2** at 298 K.

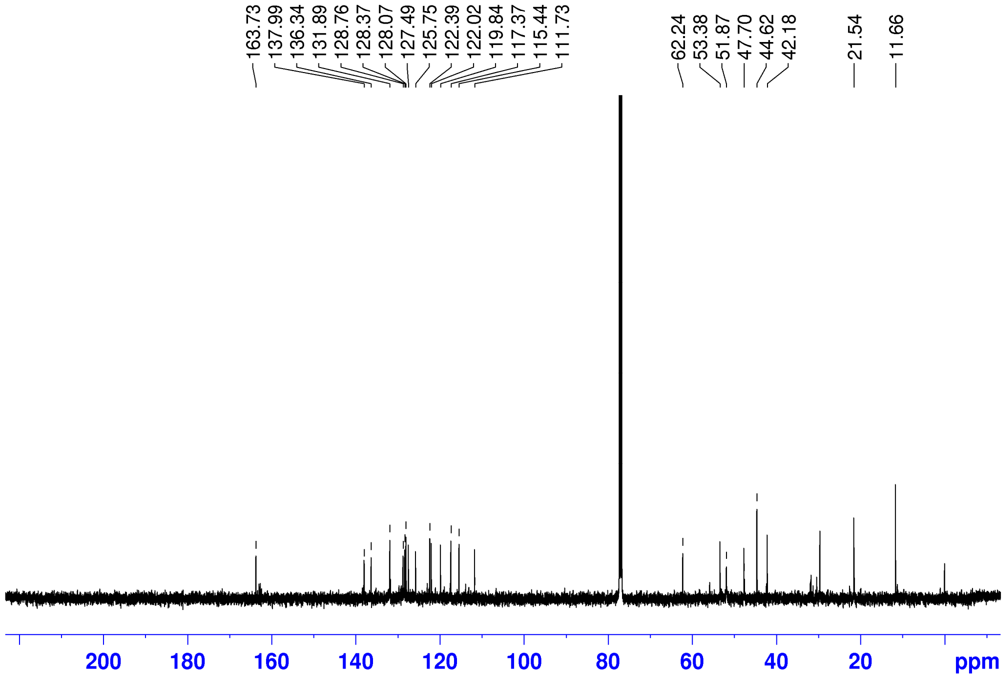

Figure S5. ^{13}C NMR ($CDCl_3$) of compound *trans*-**2** at 298 K.

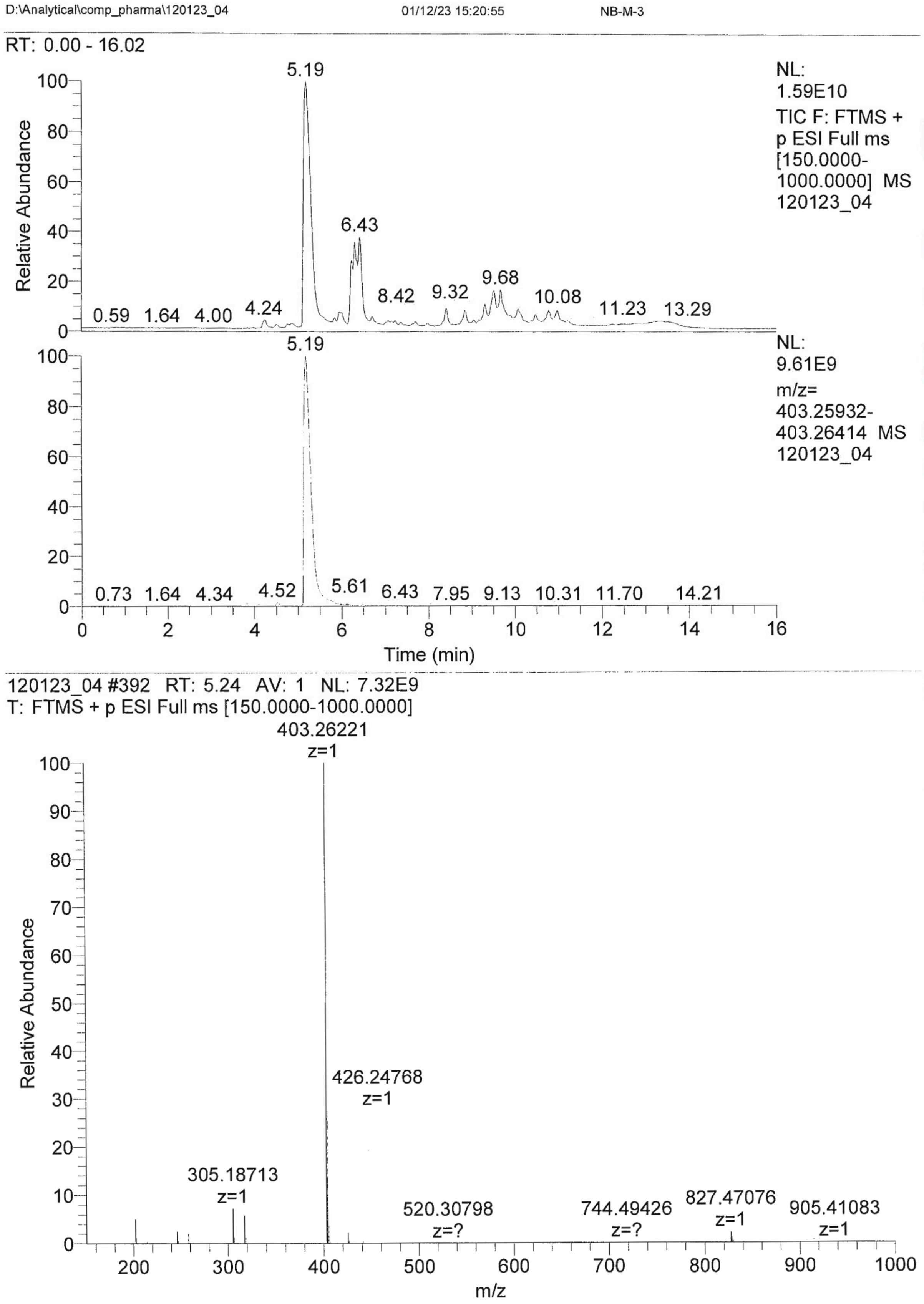

Figure S6. HRMS spectrum of compound *trans*-**2.**

General structure of compounds *trans-3* - *trans-9*

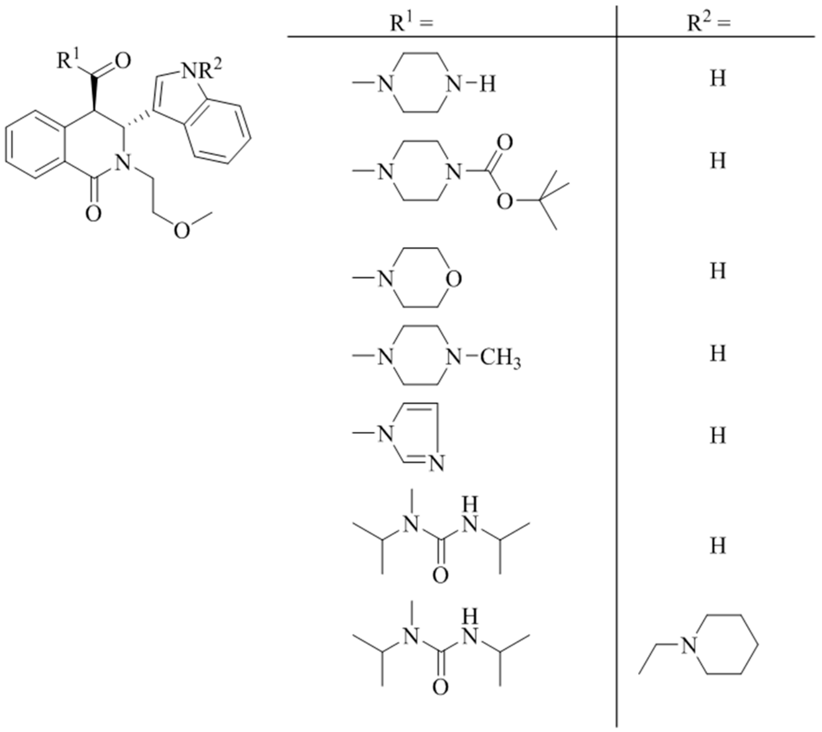

trans-**3**
trans-**4**
trans-**5**
trans-**6**
trans-**7**
trans-**8**
trans-**9**

Figure S7. General structure of compounds *trans*-**3** - *trans*-**9**
